# A novel technique of piercing ears

**DOI:** 10.4103/0970-0358.73475

**Published:** 2010

**Authors:** Satish M. Kale, Surendra B. Patil, Sumeet Jaiswal, Nishant Khare

**Affiliations:** Department of Plastic Surgery, Government Medical College, Nagpur, Maharashtra, India

Sir,

Ear piercing is done mostly for aesthetic reasons but in our country, it is practiced as a religious and cultural tradition too. The cosmetic shops and earring kiosks use hand-powered earring guns or needles to pierce ears. Plastic surgeons are many a times requested to pierce a virgin ear lobule, or they have to pierce it after repair of split ear or a post traumatic avulsed lobule. With increasing fashion of multiple earringscartilage piercing is also becoming popular. After piercing the lobule, some stent is required to place in the hole to maintain its patency for few weeks, until the hole gets epithelized. Nonmedical personnel use gold wire or plastic stud to maintain patency for such a duration of time.

**Figure 1:**
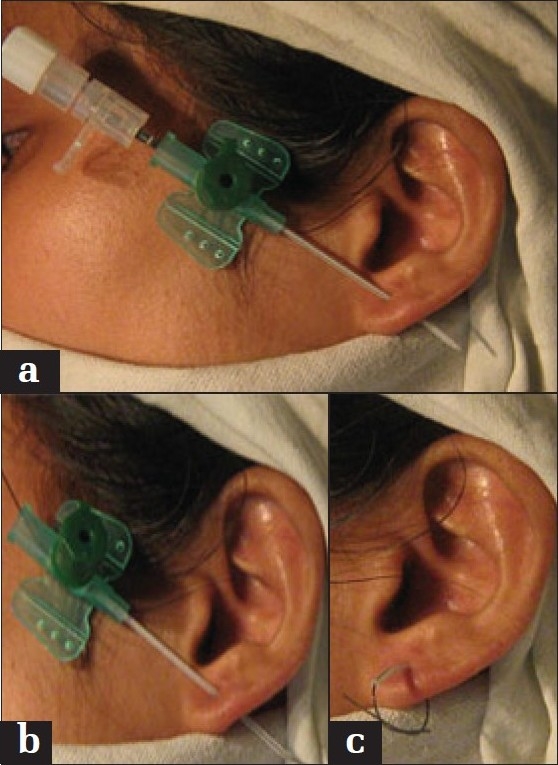
(a) Intracath inserted into lobule. (b) Stellate withdrawal and thread inserted. (c) Thread tied across plastic cannula

The stent used should be sterile and must not be displaced from its place. Under non-medical supervision, there is always a high risk of infection and other complications with this procedure.[1] Although there are varieties of ear-piercing technique described in the literature, we have devised a novel way of piercing the ear lobule.[2–4] After ink marking on proposed site of piercing and infiltrating local anesthetic agent (2% lignocaine with 1:200,000 adrenaline), a 18 guage intracath is inserted into lobule to create a hole [Figure 1a]. The stiletteof intracath is withdrawn gradually, leaving the plastic cannula in situ [Figure 1b]. The distal 1 cm of plastic cannula adjacent to the pierced ear is cut from remaining part of cannula and the injection port. A thick silk or prolene suture (1-0) is passed through lumen of cannula tube, and a knot is secured to keep tube in its place [Figure 1c]. The tube is removed after few weeks, till then the tract gets epithelized. Thus, this is a sterile, fast, safe, easy, and reliable technique for ear piercing.
